# Supplementary material for: The yeast telomerase module for telomere recruitment requires a specific RNA architecture
Source: RNA. 2018 Aug;24(8):1067–79. doi: 10.1261/rna.066696.118 (PMC6049500; doi:10.1261/rna.066696.118)
Supplement: Supplemental Material [file supp_24_8_1067__index.html]

The yeast telomerase module for telomere recruitment requires a specific RNA architecture — Supplemental Material 

# The yeast telomerase module for telomere recruitment requires a specific RNA architecture

## Supplemental Material

- Supplemental\_Figures.pdf
- Supplemental\_Legends.docx
