# Supplementary figures and images for: The yeast telomerase module for telomere recruitment requires a specific RNA architecture
Source: RNA. 2018 Aug;24(8):1067–79. doi: 10.1261/rna.066696.118 (PMC6049500; doi:10.1261/rna.066696.118)

## Supplemental Figures

Laterreur et al.; Figure S1

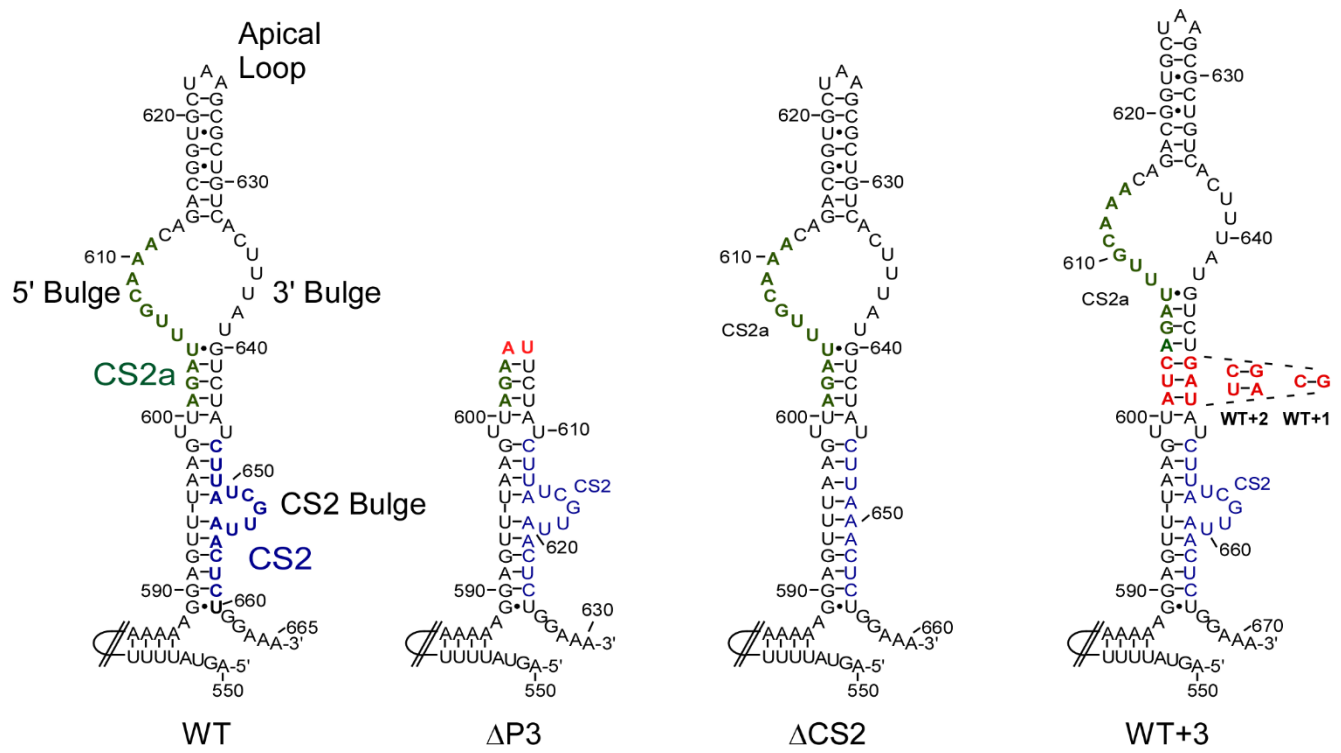

Laterreur et al.; Figure S2

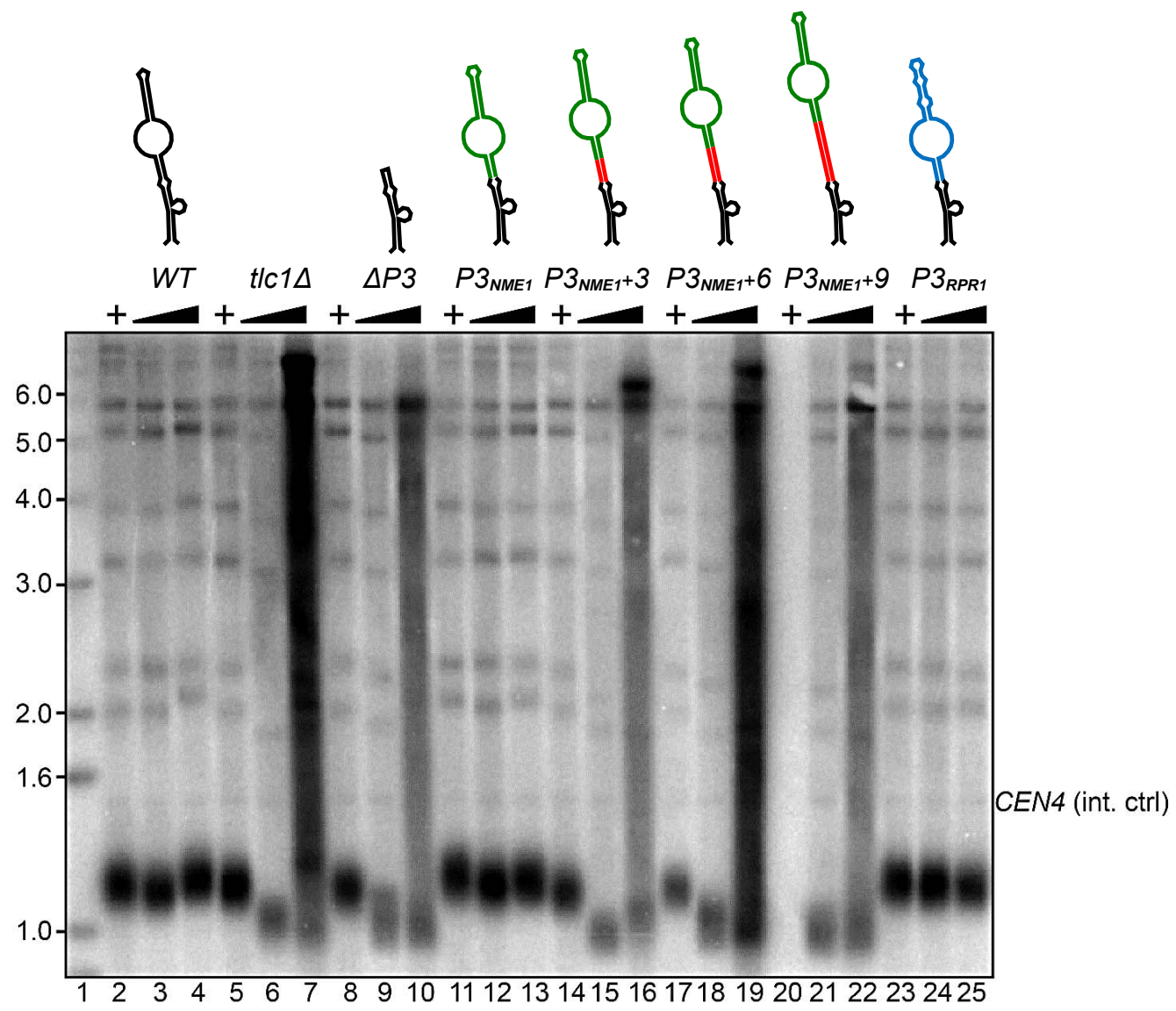

Laterreur et al.; Figure S3

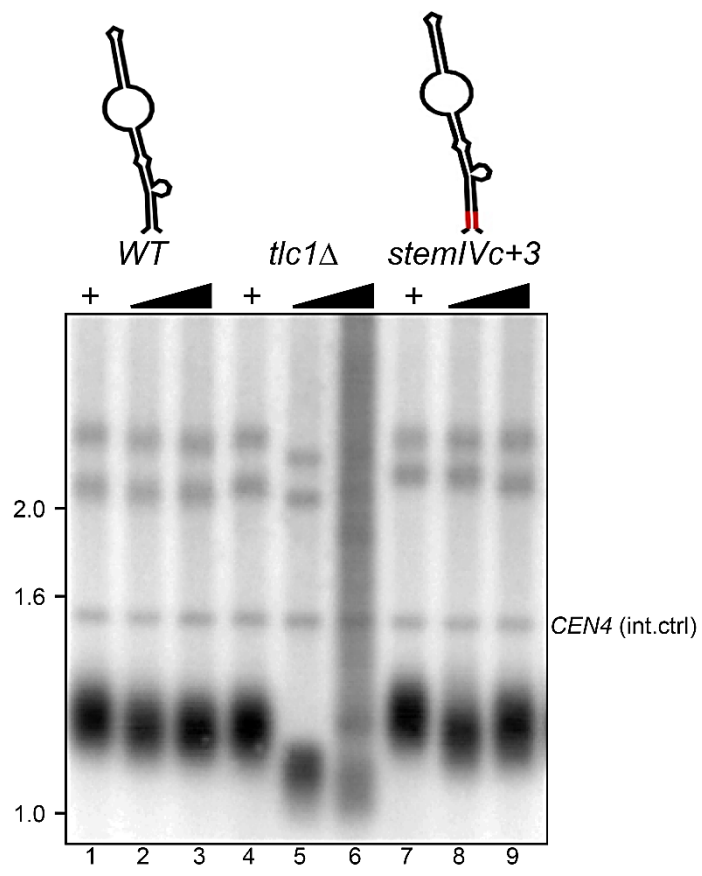

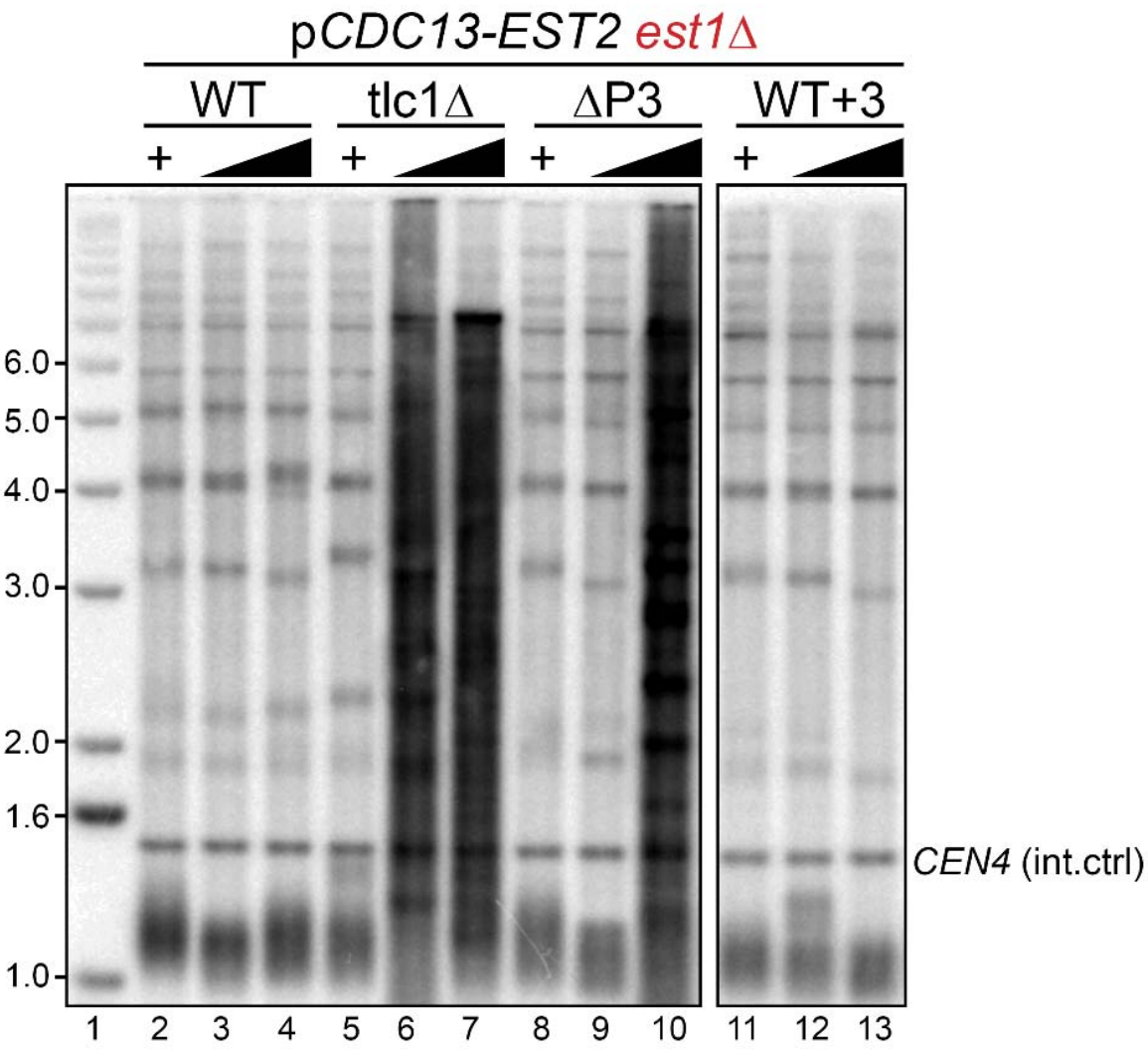

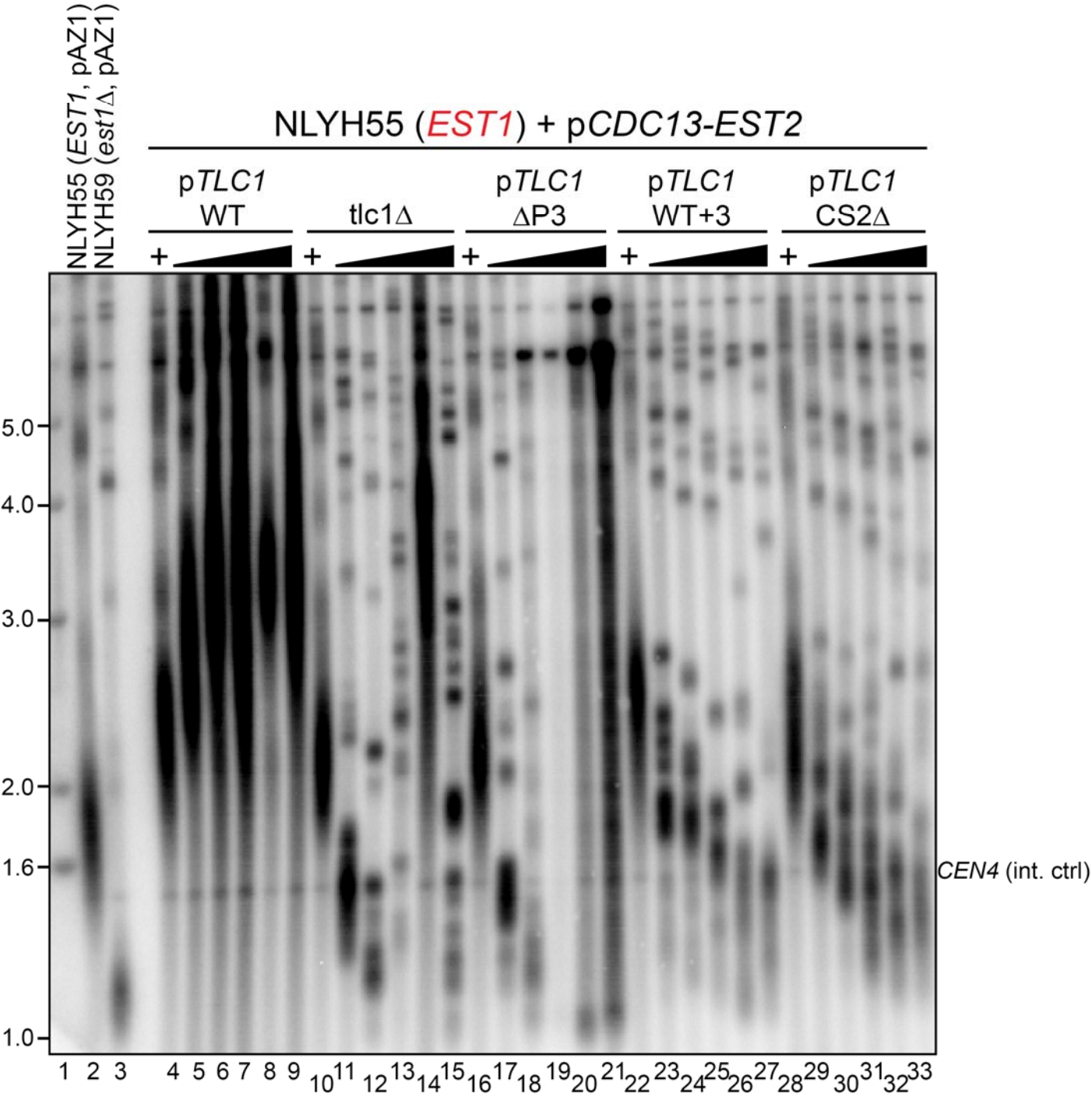

Supplement: Supplemental Material [file supp_066696.118_Supplemental_Figures.pdf]
