# Supplementary material for: The yeast telomerase module for telomere recruitment requires a specific RNA architecture
Source: RNA. 2018 Aug;24(8):1067–79. doi: 10.1261/rna.066696.118 (PMC6049500; doi:10.1261/rna.066696.118)
Supplement: Supplemental Material [file supp_066696.118_Supplemental_Legends.docx]

The yeast telomerase module for telomere recruitment requires a specific RNA architecture.

Nancy Laterreur^1^, Bruno Lemieux^1^, Hannah Neumann^1^, Jean-Christophe Berger-Dancause^2^, Daniel Lafontaine^2^ and Raymund J. Wellinger^1^*

1: Dept. of Microbiology and Infectiology
Faculty of Medicine and Health Sciences; Université de Sherbrooke
PRAC, Rm Z8-3025
3201 Rue Jean Mignault
Sherbrooke, QC, J1E 4K8

2: Dept. of Biology
Faculty of Sciences; Université de Sherbrooke
2500, boul. de l'Université
Sherbrooke Québec J1K 2R1

* Corresponding Author. Email [Raymund.Wellinger@Usherbrooke.ca](mailto:Raymund.Wellinger@Usherbrooke.ca)

Running Head: Telomerase RNA architecture of recruitment module

Keywords: Telomerase RNA, Ribonucleoproteins, Telomeres, RNA architecture

**Abstract:**

Telomerases are ribonucleoprotein (RNP) reverse transcriptases. While telomerases maintain genome stability, their composition varies significantly between species. Yeast telomerase RNPs contain an RNA that is comparatively large and its overall folding shows long helical segments with distal functional parts. Here we investigated the essential stem IVc module of the budding yeast telomerase RNA, called Tlc1. The distal part of stem IVc includes a conserved sequence element CS2a and structurally conserved features for binding Pop1/Pop6/Pop7 proteins, which together function analogously to the P3 domains of the RNase P/MRP RNPs. A more proximal bulged stem with the CS2 element is thought to associate with Est1, a telomerase protein required for telomerase recruitment to telomeres. Previous work found that changes in CS2a cause a loss of all stem IVc proteins, not just the Pop proteins. Here we show that the association of Est1 with stem IVc indeed requires both the proximal bulged stem and the P3 domain with the associated Pop proteins. Separating the P3 domain from the Est1 binding site by inserting only 2 base pairs into the helical stem between the two sites causes a complete loss of Est1 from the RNP and hence a telomerase-negative phenotype in vivo. Still, the distal P3 domain with the associated Pop proteins remains intact. Moreover, the P3 domain ensures Est2 stability on the RNP independently of Est1association. Therefore, the Tlc1 stem IVc recruitment module of the RNA requires a very tight architectural organization for telomerase function in vivo.

**Introduction:**

Inside cells, ends of linear DNA molecules incur losses during each round of DNA duplication. This occurs because of the so called “end-replication problem” which is caused by the inability of the canonical semi-conservative replication machinery to completely copy both strands of DNA (Watson 1972; Wellinger and Zakian 2012; Wu et al. 2017). Given that this problem is inherent to the structure of DNA and the way all DNA polymerases work, dividing cells of all eukaryotes with linear chromosomes are confronted with it. A cornerstone for a solution to the problem is the enzyme telomerase, which re-synthesizes one strand of the species-specific telomeric repeats at the telomeres (Greider and Blackburn 1987), while the other strand will be completed by canonical DNA polymerases (Diede and Gottschling 1999; Adams Martin et al. 2000). Telomerase is a ribonucleoprotein (RNP) enzyme that adds telomeric repeat DNA via reverse transcription (Greider and Blackburn 1987). The reverse transcriptase activity is carried out by the central and conserved protein subunit called Telomerase Reverse Transcriptase, TERT (Est2 in yeast), that is associated with an essential RNA moiety, the Telomerase RNA, TR; (Tlc1 in yeast), which provides the templating sequences (Greider and Blackburn 1989; Shippen-Lentz and Blackburn 1990; Singer and Gottschling 1994; Lingner et al. 1997; Nakamura et al. 1997). For humans, a telomeric repeat tract of minimal length is essential to maintain genome stability and critically short telomeres are associated with several genome destabilizing mechanisms, most of which are classical preludes to cancer (Maciejowski and de Lange 2017). Furthermore, the absence of telomerase in somatic cells limits the renewal capacity of cells, which eventually contributes to organismal aging (Opresko and Shay 2017). Both anti-cancer and promoting healthy aging approaches thus stand to profit from molecular insights into telomerase function.

Given the essentiality of telomeres on eukaryotic chromosomes, telomerase RNPs are found in a wide variety of organisms, including ciliates, fungi and vertebrates. While the telomerase-mediated reverse transcriptase based mechanism conceptually is the same in all of those organisms, the essential RNA moiety is remarkably different between them. Indeed, the telomerase RNAs display a high variability in virtually all possible RNA characteristics: the RNA polymerase synthesizing it, overall RNA length, type of 3’-end and particularly the primary sequence (see (Podlevsky and Chen 2016; Vasianovich and Wellinger 2017; Wu et al. 2017) for reviews). Consistent with this variability, the identity of the associated proteins in the telomerase holo-complex also varies significantly with only the conserved TERT/Est2 protein being universally recognizable. Nevertheless, commensurate with the RT-mechanism for telomeric repeat synthesis, certain structural elements in the TR/Tlc1 RNAs are highly conserved. These concern particularly regions at the core of the reverse transcriptase function: single stranded template region; a template boundary element; a distal stem-loop or three way junction element and an RNA pseudo-knot structure that is crucial for TERT/Est2 binding (Chen and Greider 2004; Egan and Collins 2012; Kuprys et al. 2013; Podlevsky and Chen 2016; Wu et al. 2017). The other, more variable areas of the RNAs are thought not to be crucial for telomerase catalytic activity *per se*, but rather for binding factors that mediate or stabilize RNA architecture and which will determine RNP assembly, transport, final localization and recruitment to telomeres (Vasianovich and Wellinger 2017; Wu et al. 2017). With the exception of the RNP in the ciliate *T. thermophila*, which contains a very short telomerase RNA, no general overview structures of telomerase RNPs have been determined (Jiang et al. 2013; Jiang et al. 2015). Therefore, the detailed three-dimensional architecture of the RNPs with larger RNAs (> 500 nts) remains to be determined. This is particularly the case for the yeast telomerase RNPs that contain what appear to be the largest telomerase RNAs.

Indeed, budding and fission yeast TRs all are over 900 nts long and can be over 2 kb, much longer than those of ciliates (around 140 to 210 nts) and vertebrates (310 to 560 nts)(Podlevsky and Chen 2016). The TR of *S. cerevisiae,* called Tlc1 (for TeLomerase Component 1), is a prime example, being 1157 nt long (Singer and Gottschling 1994; Dandjinou et al. 2004). The Tlc1 RNA is transcribed by RNA polymerase II, contains a typical Sm consensus site near the 3’-end, a 5’-trimethylguanine cap and is non-polyadenylated, like a snRNA (Vasianovich and Wellinger 2017). The predicted two-dimensional structure of the RNA, derived from phylogenetics and biochemical probing, revealed grouped together core telomerase elements associated with the RT (template, template boundary element and the pseudo-knot) and from there, three helical elements fan out (Dandjinou et al. 2004). At the end of the first are the conserved three-way junction and the actual 5’ and 3’-ends of the RNA (see Fig. 1A). The second arm terminates with a specific stem-loop structure that is important for yKu binding and which helps in nuclear retention of the RNP (Peterson et al. 2001; Gallardo et al. 2008). The distal portion for the third arm containing stem IVc on the RNA contains a bulged stem (CS2) that binds Est1 (Seto et al. 2002). In addition it also encompasses a bulged stem-loop arrangement with the conserved sequence element CS2a that is highly similar to the P3 domains of the Nme1 and Rpr1 RNAs (Fig. 1A)(Frank et al. 2000; Gunisova et al. 2009; Esakova and Krasilnikov 2010; Lemieux et al. 2016). These latter RNAs are the central and essential moieties of the highly conserved yeast RNaseMRP and RNaseP RNP enzymes (Esakova and Krasilnikov 2010). Further work showed that the Tlc1 P3-like domain associates with the Pop1/Pop6/Pop7 proteins; most likely via a similar architecture as determined for the Nme1 P3 domain in the yeast RNaseMRP (Perederina et al. 2007; Perederina et al. 2010; Fagerlund et al. 2015; Lemieux et al. 2016). Previous results also showed that a deletion of the Tlc1 P3-like domain caused a complete loss of Est1 from the telomerase RNP and Est2 binding was reduced (Lemieux et al. 2016). Given that Est1 is essential for telomerase recruitment to telomeres *in vivo*, cells that express a Tlc1 RNA without the P3-like domain behave like telomerase null cells (Laterreur et al. 2013; Lemieux et al. 2016). However, it remains unclear how the P3-like domain affects Est1 binding and how it interacts with the rest of the RNP. Interestingly, the budding yeast telomerase RNA does allow permutations of certain parts of the RNA. For example, the yKu-binding stem-loop as well as the complete stem III/stem IV arm domain can be moved to other locations in the RNA without complete loss of functionality (Zappulla and Cech 2004; Zappulla et al. 2011). Therefore, it was suggested that the yeast telomerase RNP may be quite flexible in its architecture and the underlying RNA may function like a modular scaffold (Zappulla and Cech 2006).

Given this proposed flexibility, here we investigated whether the Est1-binding element on Tlc1 (the bulged stem with the CS2 element, see Fig. 1A) could be separated from the distal P3-like element with CS2a binding the Pop1/Pop6/Pop7 proteins. Co-immunoprecipitations and assays probing *in vivo* telomerase function show that the Pop1/Pop6/Pop7 proteins bind to the P3-like element without the requirement for Est1 binding. In contrast, their presence is required for Est1 association with the RNP. Moreover, if the stem between the two binding elements was lengthened by more than one base pair (bp), Est1 association with the RNA was completely lost, but the Pop1/Pop6/Pop7 proteins remain associated with the intact distal P3-like domain. Selective 2′-Hydroxyl Acylation analyzed by Primer Extension (SHAPE) experiments show that this effect was not due to RNA misfolding in the domain. Therefore, while an intact P3-like domain on stem IV of the Tlc1 RNA is sufficient for the association of the Pop proteins, the association of Est1 has at least three requirements: i) the presence of the bulged stem CS2 site; ii) the presence of the Pop-proteins, and iii) a very specific and constrained architecture of the two sited towards each other. By extension, these results strongly predict that one of the Pop-proteins interacts directly with Est1. Furthermore, the presence of the P3-like domain and its associated proteins also stabilizes the catalytic Est2 protein on the RNP. This interaction however appears not dependent on a specific architecture and is still observed in the RNA variant that binds the Pop-proteins, but not Est1. These results therefore indicate that the 3D RNA structure on at least one sub-module of the yeast telomerase RNP is very distinct and not malleable, while the global structure can be flexible in its organization.

**Results:**

*Est1 association with the telomerase RNP requires both the P3-like domain and the bulged stem binding site on the RNA.*

Previous results showed that the distal bulged stem-loop, including the CS2a element, of stem IVc of the Tlc1 RNA is functionally interchangeable with the P3 domain of the Rpr1 and Nme1 RNAs (Fig. 1A and (Lemieux et al. 2016). Co-immunoprecipitation experiments also demonstrated that this part of the Tlc1 RNA was required for the association of the Pop6/Pop7 and the Pop1 proteins. Furthermore, telomerase RNPs that contained a version of the Tlc1 RNA without this P3-like domain also lacked Est1 (Lemieux et al. 2016). Consequently, telomerase with this variant of the Tlc1 RNA lost its ability to elongate telomeres *in vivo* (Laterreur et al. 2013; Lemieux et al. 2016). This physical proximity of the Est1 binding site occurring on the proximal bulged stem of stem IVc (with the CS2 element) and the distal P3-like domain (with the CS2a element, see Fig. 1A), raised the question whether stem IVc would define a complete subdomain of telomerase or whether the Est1 and Pop-protein associations could occur in an independent fashion. We therefore assessed protein co-immunoprecipitation with Tlc1 RNA constructs that were predicted to not bind either Est1 (CS2Δ, (Seto et al. 2002)), the Pop-proteins (ΔP3, (Lemieux et al. 2016)) or in which a short stem between the two binding sites was increased in size (WT+3 contains three extra bps between the sites, see Fig. S1). The respective RNAs also contained 10 MS2 core protein (MS2CP) binding stem-loops near the 3’-end of the RNA which, in conjunction with the expressed MS2Cp-ProA fusion protein, can be used to immunopurify the RNP (Gallardo et al. 2011; Lemieux et al. 2016). The precipitates were then analyzed for the presence of tagged proteins on Western blots (Est1, Est2, Pop1 and Pop6, Fig. 1B) as well as for Tlc1 RNA as positive control and the Nme1 RNA as negative control on Northern blots (Fig. 1C). When strains were expressing MS2-tagged WT Tlc1 RNA, all proteins are detected in the precipitates of the extracts, as expected (Fig. 1B, lane 6). In strains expressing an untagged Tlc1 RNA, no proteins (Fig. 1B, lane 7) nor RNA (Fig. 1C, lane 5) are immunoprecipitated, again as expected. Consistent with previous results, immunoprecipitates of tagged CS2Δ-Tlc1 RNA failed to include the Est1 protein (Seto et al. 2002), but the Est2, Pop1 and Pop6 proteins do remain associated with the RNP (Fig. 1B, lane 10). On the other hand, the ΔP3 RNA lost the Pop1/Pop6 association as well as Est1, but did retain some Est2 (Fig. 1B, lane 8). Remarkably, the WT+3 Tlc1 RNA only lost Est1 association, all other proteins appear bound to it as to the WT RNA (Fig. 1B, lane 9). These results indicate that the Pop1 and Pop6 proteins, plus presumably the Pop7 protein, associate with the distal P3-like domain independently of Est1 binding. On the other hand, these data show that the proximal bulged stem around the CS2 element alone is not sufficient for stable Est1 association.
In order to verify whether these co-immunoprecipitation results with native RNPs had functional repercussions on telomerase function *in vivo*, telomere maintenance was monitored in cells that expressed the variant Tlc1 RNAs (Fig. 2). As shown before, the ΔP3 Tlc1 RNA caused telomeric DNA losses and after a number of generations, the patterns of the terminal restriction fragments in such strains became very poorly defined. This is the expected and classical phenotype of survivor cells that lack telomerase altogether and replenish telomeric repeats via recombination (Fig. 2A, lanes 4-9). A single bp insertion into the short stem between the bulged stem CS2 and the P3-like domain was tolerated and cells expressing this variant of *tlc1* harboured wt telomeres (Fig. 2A, lanes 10-12, Fig. S1 for construct). However, insertion of two bps or more completely abolished *in vivo* telomerase activity (Fig. 2A, lanes 13-18; Fig. 2B, lanes 7-15). This effect is not due to a sequence specific effect of the inserted bases. The Tlc1 P3-like sequence can be replaced with the P3 domain of the Nme1 RNA or the P3 domain of the Rpr1 RNA without any effect on telomere maintenance (Fig. 1A, lanes 19-21; Fig. S2A, lanes 11-13 and 23-25; (Lemieux et al. 2016)). Yet, inserting either of these two P3 elements does change the primary sequence composition of the short stem between the bulged stem and the P3 element. In this case again however, inserting three bps between the bulged stem CS2 element and the P3 domain from the Nme1 RNA completely abolished telomerase activity *in vivo* (Fig. 2A, lanes 22-24), as did adding up to nine base pairs (Fig. S2A, lanes 14-22). Furthermore, inserting more bps into the same place in the WT Tlc1 never allowed a recovery of *in vivo* functionality, even if in theory with 12 bps a full helix turn and perhaps more was achieved (Fig. 2B; (Arias-Gonzalez 2014). These results underscore the requirement for a very tight organization along the stem IVc. As a control, we also tested whether inserting additional bps at the base of stem IVc had any effect on telomerase activity (Fig. S3). Inserting three bps proximal of the bulged stem CS2 site, in effect lengthening the stem IVc overall at its base, had no discernible effect on telomere length maintenance *in vivo* (Fig. S3, lanes 7-9). Taken together, these results suggest that stem IVc with the P3-like domain and the Est1 binding site is a tightly organized module in the yeast telomerase RNP. While the stem can be lengthened at its base without any measurable effect on telomerase function, it is very sensitive to perturbations within.

We considered the possibility that inserting three base pairs in a sensitive area of the RNA could alter the overall local RNA organization and hence cause the loss of Est1 independently of the distal P3-like domain. While mFold predicted two-dimensional structures that were consistent with an intact bulged stem CS2 area and a native P3-like domain folding, we probed the structures adopted by the various variants of the stem IVc RNAs by SHAPE *in vitro*. As shown previously, such SHAPE analyses on the WT stem IVc RNA yield nucleotide reaction patterns that are in complete agreement with the predicted structures (Fig. 3 WT and (Laterreur et al. 2013)). Furthermore, *in vivo* DMS probing of this part of the RNA also yielded results that are consistent with this overall arrangement (Forstemann and Lingner 2005). The RNA construct with the three base pairs inserted into the stem, WT+3, as well as the CS2Δ RNA construct yielded base reactivities that overall also are entirely consistent with the predicted structures (Fig. 3). Altogether, these results therefore are entirely consistent with the conclusion that for the WT+3 construct, loss of Est1 from the RNP was not due to the inserted bases in the RNA nor was it due to local misfolding of the RNA and a loss of the CS2 bulge for example.

*Pop1 association with the RNP occurs on the P3 domain and stabilizes Est2 binding.* Previous results showed that a tagged Pop1 protein can be used to immunoprecipitate active telomerase and that recombinant Pop1 stimulated telomerase activity *in vitro* (Lemieux et al. 2016). However, it remained possible that the Pop1 association with telomerase occurred via multiple interactions with the RNA and other proteins, as it does on the RNaseP/MRP RNPs (Esakova and Krasilnikov 2010; Fagerlund et al. 2015). We therefore analyzed immunoprecipitated telomerase that were obtained with an anti-HA antibody and extracts from cells that expressed an HA tagged Pop1 combined with the various Tlc1 RNAs described above (Fig. 4A; top panels: Western blots). Co-immunoprecipitating Tlc1 RNAs or Nme1 RNA (as positive control) were detected on Northern blots (Fig. 4A; bottom panels: Northern blot). HA-Pop1 immunoprecipitated the wt Tlc1 RNA, as expected, as well as the CS2Δ Tlc1 RNA (Fig. 4A, lanes 3 and 6). Moreover, the WT+3 Tlc1 RNA also was efficiently precipitated, consistent with the results obtained with the co-immunoprecipitation experiments reported above (Fig. 4A, lane 5; Fig. 1). However, there was no detectable ΔP3 Tlc1 RNA in the precipitates yet the positive control Nme1 RNA was readily detected (Fig. 4A, lane 4). The Myc-tagged Est2 protein followed that pattern as well: it was only detectable in the immunoprecipitates when a complete P3-like domain was present on the Tcl1 RNA (WT Tlc1 RNA, CS2Δ RNA and the WT+3 RNA) but not with the ΔP3 Tlc1 RNA (Fig. 4A). Actual telomerase activity followed the presence or absence of Est2 in the precipitates, as would be predicted (Fig. 4B). These experiments therefore establish that Pop1 binding on the Tlc1 RNA does require the P3-like domain. Moreover, its binding appears sufficient for supporting full telomerase activity in the *in vitro* assay, even if the RNA contains mutations in the stemIV domain (the WT+3 or the CS2Δ RNAs).

In order to verify telomerase activity *in vivo*, we introduced the above variants of the Tlc1 RNA into strains in which the Est2 protein is directly fused to the telomere-binding protein Cdc13. This fusion protein mediates a forced recruitment of telomerase to telomeres allowing telomeric repeat maintenance even in the absence of endogenous Est1 (Evans and Lundblad 1999). This setup therefore allowed an examination of *in vivo* telomerase activity independently of Est1. In cells without the endogenous Est1 protein (*est1Δ-*cells) that express that Cdc13-Est2 fusion protein, the Tlc1 RNAs with the CS2Δ or the WT+3 mutations allowed telomere maintenance in a fashion that is very similar to the wt Tlc1 RNA (Fig. 5A, compare lanes 5-9, with lanes 23-27 and 29-33; also Fig. S4). Upon long-term culturing of these cells, telomeres gradually lengthen beyond the wt length, an effect that was not previously observed (Fig. 5A, lanes 6-9). On the other hand, just like in the situation of a complete absence of the Tlc1 RNA (*tlc1Δ-*cells), the ΔP3 Tlc1 RNA could not support telomere maintenance: the mechanism switched to a recombination based elongation and the terminal restriction fragment patterns changed to one typically seen in such survivor cells (Fig. 5A lanes 11-21, Fig. S4 lanes 5-10; (Wellinger and Zakian 2012)). In cells in which Est1 was expressed, conceptually similar results were obtained for the mutant RNAs: the ΔP3 Tlc1 RNA caused telomere loss as observed in cells lacking Tlc1 RNA altogether (*tlc1Δ-*cells; Fig. 5B, compare lanes 9-13 with lanes 14-18, and Fig. S5 after 300 generations). In contrast, cells that contained the WT+3 RNA or the CS2Δ RNA eventually maintained telomeres at about 400 to 500 bps (Fig. 5B, lanes 22-23 and 27-28, and see Fig. S5, lanes 26-27 and 32-33 for long term culturing), similar to what is seen in the cells without Est1 (est1Δ-cells; Fig. 5A lanes 7-9 after 300 generations). These data therefore directly demonstrate that the WT+3 and the CS2Δ Tlc1 RNAs both support telomerase activity *in vivo*. Furthermore, they show that the ΔP3 Tlc1 RNA does not support enough telomerase activity to maintain telomeres *in vivo*, and that this effect is independent of the loss of Est1. Hence, the absence of the P3-like domain in the ΔP3 Tlc1 RNA causes a loss of telomerase catalytic activity that is independent from the loss of the Est1 protein mediated recruitment.

Finally, these results also show that Est1 plays a direct role in telomere elongation dynamics. For example, in the presence of a wt Tlc1 RNA and the Est1 protein, telomeres are over-elongated to reach equilibrium lengths of over 2 kb, as previously described (Fig. 5B, lane 8; Fig. S5, lanes 8, 9; (Evans and Lundblad 1999)). In contrast, in all three situations in which Est1 is lost from the RNP, the eventual equilibrium length of telomeres is much shorter. First, with both Tlc1 variants that lose Est1-association (CS2Δ and WT+3), even if Est1 is expressed, telomere length stabilizes at about wt length (Fig. 5B, lane 23 and 28; Fig. S5 lanes 26-27 and 32-33). These sizes are very comparable to what is observed in *est1Δ-*cells that express a wt Tlc1 RNA (see Fig. S4 lanes 3-4), again essentially a telomerase RNP without Est1. These results therefore show that Est1 association with the telomerase RNP influences elongation characteristics of the enzyme in a way that is independent from its function in telomerase recruitment.

**Discussion:**

The large telomerase RNAs found in yeast species have been described as providing flexible scaffolds for protein tethering to the RNP (Zappulla and Cech 2006). The strongest evidence supporting this hypothesis is provided by experiments showing that large parts of the RNA can be moved to different positions in the RNA without loss of telomerase function (Zappulla and Cech 2004; Zappulla et al. 2011). The overall idea then was that Protein-RNA interactions were required for overall tethering but that the final organization of the RNP could be heterogeneous and achieved in various ways (Zappulla and Cech 2006). However, more recent data showed that at least for the short RNA telomerase from T. *thermophila*, there is a specific overall structure of the RNP which is strongly molded by sub-modules comprising multiple proteins (Jiang et al. 2013; Jiang et al. 2015). The general principles of the tridimensional organization of the telomerase RNPs therefore still need to be worked out.

Here we present results showing that in budding yeast, the RNA domain required for telomerase recruitment to telomeres does not allow much architectural change. In particular, the two main protein-binding sites on stem IVc of the RNA appear to closely interact with each other and adding more than 1 bp in the short stem between them causes a complete loss of the Est1 protein (Fig. 1). As a consequence, these insertions also caused a telomerase-negative phenotype *in vivo* (Fig. 2). These findings are most easily explained if there was a direct interaction interface between Est1 and any of the three Pop-proteins associated with the distal P3-like domain. Insertion of 2 bps into the intervening stem could cause the distal P3-like domain to be rotated by up to 65° with respect to the Est1 protein and also introduce some additional distance between them. The data do show that these insertions still allow the Pop6/Pop1 proteins, plus presumably the Pop7 protein, to associate with the distal P3-like domain, suggesting that the complete P3-domain will bind the Pop-proteins independently of any other proteins. We note that these *in vivo* results are completely consistent with previously established *in vitro* binding assays (Lemieux et al. 2016). However, the Est1 protein is lost from the RNP in the insertion mutants. We do not yet have evidence for the predicted interaction surface on any of the proteins and therefore, the possibility of independent associations of Est1 and the Pop-proteins with the RNA without any interactions between them cannot completely be excluded. However, this latter possibility predicts that Est1 contacts the RNA somewhere distal to the CS2 bulged stem. A number of observations strongly disfavour this possibility: 1) the sequence of the short stem between the Est1 binding site and the P3 domain (nts 600-604:640-644 in Fig. 1A, see also Fig. S1) can be changed without loss of function. For example, it differs on the RNA of very closely related Saccharomyces species or introduction of the P3-elements of the Nme1 or Rpr1 RNAs also cause that sequence to change, yet telomerase in all these cases remains fully functional *in vivo* (Dandjinou et al. 2004; Lemieux et al. 2016). The insertion of 3 bps into that stem (see Fig. S1) does not alter the structure of the RNA in this area, as assessed by SHAPE (Fig. 3) and therefore could not by itself be the reason for a loss of Est1. Conversely, a Tlc1 variant in which the more distal CS2a sequences were mutated but the short stem between CS2 and CS2a remained WT caused a complete loss of telomere maintenance (Gunisova et al. 2009; Lemieux et al. 2016). Therefore, the loss of Est1 from the WT+3 construct cannot be due to a loss of an Est1 binding site at the place of insertion. 2) The crystal structure of the Po6/Pop7 proteins bound to the P3 domain RNA shows extensive protein-RNA interactions, particularly for the bulged single stranded RNA nucleotides of the CS2a element and the opposing bulge (Perederina et al. 2010). Moreover, our previous data strongly suggest that the Pop6/Pop7 - P3-like RNA complex is a constitutive unit on the telomerase RNP (Lemieux et al. 2016). Therefore, direct contacts of Est1 with these single-stranded bases are highly unlikely. 3) The above would leave interactions of Est1 with the terminal stem loop on stem IVc (nts 615-630 in Fig. 1A). However, that stem-loop is not absolutely essential for telomerase activity as it can be removed from the Tlc1 RNA and telomeres are still maintained (Lemieux et al. 2016). In addition, the Est1 protein would have to wrap around and over the Pop6/Pop7 proteins to reach these distal nucleotides, which is improbable by itself. For all of these reasons, the simplest and most likely scenario explaining all the data is that Est1 interacts directly with one or more of the Pop-proteins. Given the strength and solidity of the Pop6/Pop7 - P3-RNA complex, it might not be very flexible and therefore, its orientation with regard to the Est1 binding site on the CS2 site be relatively restrained. Hence, introducing additional nucleotides between the CS2 and the CS2a sites would cause a rotational change and the loss of Est1.

In addition, the results presented here include *in vivo* evidence that the Est1 protein makes a contribution to catalytic telomerase activity that is independent from its recruitment function. Previous genetic and biochemical experiments already suggested that Est1 contributes to telomere maintenance in ways that are independent from its interactions with Cdc13, and hence recruitment (Evans and Lundblad 2002; Wu and Zakian 2011). Our data show that if telomerase recruitment is bypassed via expression of a Cdc13-Est2 fusion protein, final steady state telomere lengths are different when Est1 is associated with telomerase (Fig 5B, Tlc1 wt; and Fig. S5, Tlc1 wt) as compared to when it is not associated with telomerase (Fig. 5B, Tlc1-CS2Δ or Tlc1-WT+3; Fig. S5, Tlc1-CS2Δ or Tlc1-WT+3). Note that in all these experiments, a wtEst1 protein is expressed and present in the cells and the results therefore cannot be ascribed to a lack of the Est1 protein in general with pleiotropic effects or an inability to interact with Cdc13. In addition, the terminal telomere length phenotype in these cells with the Tlc1-CS2Δ or Tlc1-WT+3 RNAs is also different from the one observed in cells lacking the Tlc1 RNA altogether (Fig 5B and Fig. S5). In the latter, no telomerase is present and telomere maintenance eventually switches to a recombination based mechanism. Taken together, telomere elongation in the cells expressing the Cdc13-Est2 fusion protein is telomerase dependent but Tlc1 RNA mutants, which are unable to associate with Est1 mediate a much reduced elongation as compared to wt Tlc1 RNA. Therefore, these results directly show that, at least in the Cdc13-Est2 fusion situation, Est1 also contributes to telomere elongation during the reaction cycle and not only via its interaction with Cdc13.

Finally, the results obtained with the WT+3 construct demonstrate that a functional P3 domain with the associated Pop-proteins does stabilize the Est2 protein on the RNP. In contrast to the Est1 interaction, this stabilization is not affected by RNA sequence modifications on stem IVc as long as they leave the P3-like domain intact (Fig. 4). Therefore, the actual orientation of distal P3-like domain on the RNP is flexible with respect to Est2, but not with respect to Est1.

In summary, the results presented here demonstrate that the stem IVc domain of the budding yeast telomerase RNA Tlc1 is a very tightly organized module that minimally comprises the proper RNA structure, the Est1 and the Pop1/Pop6/Pop7 proteins. Furthermore, in order for a functional telomerase RNP to assemble, Est1 most likely directly interacts with the Pop-proteins. Next, while previously this domain was thought to be required exclusively for telomerase recruitment to telomeres, the combined evidence (Fig. 6 in (Lemieux et al. 2016) and Fig.1 here) suggest that the distal P3-like domain also serves to stabilize Est2 on telomerase. Finally, using specific constructs that bypass the requirement for the recruitment function of Est1, we established that Est1 contributes to the telomere elongation reaction directly. Further structural and functional studies will be needed to describe what this contribution is and how it is integrated in the general telomere maintenance mechanisms.

**Material and Methods:**

**Strains and plasmids**

A list of all strains with the associated genotypes and plasmids with general properties used in this study can be found in Tables 1 and 2. Strains EBYH03, NLYH55 and NLYH59 were used for telomere length analyses of the different *TLC1* variants. The strains were transformed with the various *TLC1* plasmids and pAZ1. After growth on FOA-TRP-LEU plates to select for the loss of the WT copy of *TLC1*, cells were passaged for the indicated generations on selective media. Strains NLYH55 and NLYH59 contain pVL1107 (*CDC13-EST2*). For western and northern blots, as well as telomerase activity assays, the strain YVL3493 was used as a base to create NLYH125, NLYH128 and NLYH494. *HA3-POP1* was integrated by linearizing pNL09 with SpeI, grown on Sc-URA, and then on 5-FoA to select for clones that have lost the plasmid backbone. Clones were screened for HA_3_-Pop1 expression by western blot. Tagging of *POP6* with the HA3 epitope in NLYH125 was achieved by PCR-based gene targeting using the pFA6a-3HA-KMX plasmid and this generated strain NLYH128 (Bahler et al. 1998). Strain NLYH494 was created as described in (Lemieux et al. 2016).

Mutagenesis of p*TLC1TRP* was achieved by two-step directed PCR mutagenesis to generate pTLC1-WT+1, pTLC1-WT+2, pTLC1-WT+3, pTLC1-WT+6, pTLC1-WT+9, pTLC1-WT+12 and the P3-replacement constructs. The different PCR fragments were used to replace a NcoI-NsiI segment of *TLC1*. The clones were then verified by sequencing; see specific inserted sequences in the plasmid descriptions in Table 2. pTLC1-10xMS2-[WT+3] and pTLC1-10xMS2-CS2Δ were generated by Gibson assembly (Gibson et al. 2009). pNL09 was generated by 3-fragment Gibson assembly in order to clone a fragment containing 715 bps of the *POP1* promoter, the 3HA epitope and 2625 bps of the *POP1* genomic locus into the pRS306 plasmid.

**Protein extracts for co-immunoprecipitations**

Total protein extracts were prepared as described in (Laterreur et al. 2013). Briefly, 500 mL of cells grown to an OD660 of 1.0 were pelleted, washed once with cold water and once with TMG buffer (10 mM Tris-HCl pH 8.0, 1 mM MgCl2, 10% glycerol) supplemented with 200 mM NaCl. The cell pellets were then frozen in liquid nitrogen and lysis was performed by grinding the pellets in the presence of pieces of dry ice in a standard coffee mill (Krups). The cell powder was thawed on ice and 1 pellet volume of TMG (200 mM NaCl, 0.1 mM DTT, 0.2% Triton X-100, 0.2% NP40 and protease inhibitors) was added. Immunoprecipitations were done on 3 mg of total proteins adjusted to 0.5% Tween-20 and to which 40 units of RNasin (Promega, USA) was added per mL of extract. To study TLC1 variants binding to the telomerase, Pop1 and Pop6 proteins, the adjusted extracts containing MS2-ProA were supplemented with 50 µL rabbit IgG (Sigma) coated magnetic beads (Dynabeads antibody coupling kit, Life technologies). Following an incubation of 3-4 hours at 4°C, the beads were washed twice with 0.5 mL TMG2 (200 mM NaCl, 0.1 mM DTT, Complete mini EDTA free protease inhibitor (Roche) and 0.5 % Tween-20) and twice with 0.5 mL TMG1 (0.1 mM DTT, protease inhibitor and RNasin added at 40U per 0.5 mL of TMG). The washed beads were resuspended in 50 µL TMG3 (0.5 mM DTT, protease inhibitor and 40U of RNasin). For the analysis of telomerase activity in TLC1 variants, protein extracts containing HA_3_-Pop1 were prepared as mentioned above but were supplemented with 50 µL anti-HA conjugated magnetic beads (Life Technologies). The beads were washed and resuspended as described.

**Western blots**

After the removal of TMG3 from IP beads, input and IP samples were mixed with 2X Laemmli loading buffer. The proteins were then denatured for 5 min at 98ºC, separated on either 8% or 15% SDS-PAGE gels. For Pop6-HA_3_ binding analyses, the samples were run on 15% SDS-PAGE gels. For HA_3_-Pop1, Est1-Myc_12_ and FLAG_3_-Myc_12_-Est2 visualization, inputs and IP samples were mixed with 2X Laemmli loading buffer, heated and separated on 8% SDS-PAGE gels. After transfer onto Hybond-ECL nitrocellulose membranes (GE Healthcare), membranes were blocked in 5% milk/PBS-Tween and incubated with a 1:10,000 dilution of mouse monoclonal anti-ProA (Sigma), or with a 1:5,000 rabbit polyclonal anti-ProA (Sigma), or a 1:1,000 dilution of mouse anti-HA (Roche) antibodies. Secondary antibodies were HRP-conjugated sheep anti-mouse IgG and donkey anti-rabbit IgG (GE Healthcare), diluted 1:5,000 in 1% milk/PBS- T. Blots were visualized with a LAS-4000 (GE Healthcare).

**RNA-IP and northern blot analyses**

RNA-IP and northern blot analyses were performed as described in (Lemieux et al. 2016). Briefly, following immunoprecipitation of total protein extracts, TMG3 was removed from the beads and 400 µL of LETS buffer (10 mM Tris-HCl pH 7.5, 100 mM LiCl, 10 mM EDTA pH 8.0, 0.2% SDS) were added to beads, input and flow-through fractions. For total RNA extractions, 10 mL of yeast cells from TLC1-variants strains were grown to OD 0.8, collected, washed, resuspended in 400 µL LETS Buffer and lysed by glassbeads. Samples (RNA-IP and total RNA extracts) were then extracted twice with phenol/chloroform/isoamyl alcohol (25:24:1) and once with chloroform/isoamyl alcohol (24:1). After the addition of NaOAc (final conc. 150 mM) and glycogen (50 µg), the RNA was precipitated with 2 volumes of cold 100% ethanol. Samples were then washed, dried and resuspended in 20 µL of nuclease-free water. To analyse RNAs, either 10 µg of total RNA extracts or 5 µL of input, IP and FT were mixed with 1X MOPS (pH 7), 3.7% formaldehyde, 45% formamide and 1X RNA dye. RNAs were separated in a 1.2% agarose-1X MOPS (pH 7)-2% formaldehyde gel by electrophoresis. The gel was transferred to a Hybond N+ membrane (GE Healthcare). Following UV-crosslinking, the membrane was pre-hybridized and hybridized with a radiolabeled TLC1-specific probe of 694 bps (NcoI-NsiI digestion of pTLC1-TRP) and a 5’-end labeled NME1-specific oligonucleotide probe (5’-GCAATAGAGGTACCAGGTCAAGAAG-3’). Visualization and quantification were performed using a Typhoon FLA9000 apparatus and the Quantity One software.

**Southern blot for telomere length analysis**

After selection for cells that had lost pAZ1(TLC1 WT) on 5-FOA plates and consecutive streaking on selective media up to the desired generations, cells from indicated passages were grown in liquid media. Genomic DNA was extracted, digested with XhoI, subjected to agarose gel electrophoresis (0.75% agarose), transferred to a nylon membrane and hybridized to a 300 bps fragment containing 280 bps of telomeric repeats derived from pYLPV (pCT300) (Wellinger et al. 1993) and a 447-bps PCR fragment amplified from the *CEN4* genomic locus (primers: 5’-ATGCTGTCTCACCATAGAGAAT-3’ and 5’-CGCTCCTAGGTAGTGCTTT-3’). Probes for hybridization were obtained by random priming labelling procedure (Feinberg and Vogelstein 1983). Data were visualized and analyzed using a Typhoon FLA9000.

**Selective 2’Hydroxyl Acylation analyzed by Primer Extension (SHAPE)**

Wild Type and mutant (P3+3, deltaCS2, deltaP3) RNAs were T7-transcribed from a DNA template with a RT primer binding site at the 3’ end (CGCGGAGCTAAAGAGAAGCGGAAG), essentially as described before (Laterreur et al. 2013). For the SHAPE reactions, 1 pmol of RNA molecules were heated at 75ᵒC, slow cooled at room temperature and incubated at 37ᵒC for 10 min with folding buffer (100 mM Tris-HCl ph 7.5, 100 mM KCl, 10 mM MgCl_2_). Then, the samples were reacted with either 10mM N-methylisatoic anhydride (NMIA) or DMSO for 30 min at 37ᵒC. Reverse transcription reactions were performed with InVitrogen SuperScript-III RT according to the supplier’s protocol. Gels were exposed on GE phosphorScreen and scanned. Nucleotide reactivity was analysed by QuantityOne. After background adjustment, the reactivity ratio between treated (10 mM NMIA) and untreated (DMSO) molecules was calculated for each position. To normalize the reactivity pattern, each nucleotide was compared to the highest reactivity value (A21-WT), thus giving a ratio from 0 to 1, where a value between 0.2 and 0.4 was considered significantly reactive and values above 0.4 were considered highly reactive.

**Telomerase assay**

Yeast telomerase activity assays were carried out as previously described (Laterreur et al. 2013) with 10% of the IP-beads. Briefly, the extension of a telomeric primer (5’-TAGGGTAGTAGTAGGG-3’) in the presence of radiolabeled [α-^32^P] dGTP was monitored to determine telomerase activity. Extension products were separated on 18% polyacrylamide/8M urea electrophoresis gels and visualized using a Typhoon FLA9000 apparatus (GE Healthcare). As internal controls, 12-nt (5’- TTAGGGTTAGGG-3’) and 16-nt (5’-TAGGGTAGTAGTAGGG-3’) primers were 5’-end labeled and added to the samples before ethanol precipitation at 2000 cpm/µL. Quantification was performed using the Quantity One program (Bio Rad) by adding telomerase extension products signals and dividing them by the loading control signals to give Relative Telomerase Activity (RTA) ratios with respect to wildtype conditions.

**Acknowledgements:**

We thank members of the Wellinger lab for their help and A. Krasilnikov (Penn State Uinversity, University Park, USA) for discussions, very helpful suggestions and a critical reading of the manuscript. We also thank V. Lundblad (The Salk Institute, San Diego, USA) for tools such as strains and plasmids, and P. Chartrand (Université de Montreal, Montreal, Canada) for input on the manuscript. This work was supported by grants of the Canadian Institutes of Health Research (MOP97874 and FDN154315 to RJW; PJT153205 to DL), as well as the Center for Research on Aging (CdRV Sherbrooke), and the Center for RNA Biology of the Université de Sherbrooke. RJW holds the Canadian Research Chair in Telomere Biology.

**References:**

Adams Martin A, Dionne I, Wellinger RJ, Holm C. 2000. The function of DNA polymerase alpha at telomeric G tails is important for telomere homeostasis. *Mol Cell Biol* **20**: 786-796.

Arias-Gonzalez JR. 2014. Single-molecule portrait of DNA and RNA double helices. *Integrative biology : quantitative biosciences from nano to macro* **6**: 904-925.

Bah A, Bachand F, Clair E, Autexier C, Wellinger RJ. 2004. Humanized telomeres and an attempt to express a functional human telomerase in yeast. *Nucleic Acids Res* **32**: 1917-1927.

Bahler J, Wu JQ, Longtine MS, Shah NG, McKenzie A, 3rd, Steever AB, Wach A, Philippsen P, Pringle JR. 1998. Heterologous modules for efficient and versatile PCR-based gene targeting in Schizosaccharomyces pombe. *Yeast* **14**: 943-951.

Beeler T, Gable K, Zhao C, Dunn T. 1994. A novel protein, CSG2p, is required for Ca2+ regulation in Saccharomyces cerevisiae. *J Biol Chem* **269**: 7279-7284.

Chen JL, Greider CW. 2004. An emerging consensus for telomerase RNA structure. *Proc Natl Acad Sci U S A* **101**: 14683-14684.

Dandjinou AT, Levesque N, Larose S, Lucier JF, Elela SA, Wellinger RJ. 2004. A phylogenetically based secondary structure for the yeast telomerase RNA. *Curr Biol* **14**: 1148-1158.

Diede SJ, Gottschling DE. 1999. Telomerase-mediated telomere addition in vivo requires DNA primase and DNA polymerases alpha and delta. *Cell* **99**: 723-733.

Egan ED, Collins K. 2012. Biogenesis of telomerase ribonucleoproteins. *RNA* **18**: 1747-1759.

Esakova O, Krasilnikov AS. 2010. Of proteins and RNA: the RNase P/MRP family. *RNA* **16**: 1725-1747.

Evans SK, Lundblad V. 1999. Est1 and Cdc13 as comediators of telomerase access. *Science* **286**: 117-120.

-. 2002. The Est1 subunit of Saccharomyces cerevisiae telomerase makes multiple contributions to telomere length maintenance. *Genetics* **162**: 1101-1115.

Fagerlund RD, Perederina A, Berezin I, Krasilnikov AS. 2015. Footprinting analysis of interactions between the largest eukaryotic RNase P/MRP protein Pop1 and RNase P/MRP RNA components. *RNA* **21**: 1591-1605.

Feinberg AP, Vogelstein B. 1983. A technique for radiolabeling DNA restriction endonuclease fragments to high specific activity. *Anal Biochem* **132**: 6-13.

Forstemann K, Lingner J. 2005. Telomerase limits the extent of base pairing between template RNA and telomeric DNA. *EMBO Rep* **6**: 361-366.

Frank DN, Adamidi C, Ehringer MA, Pitulle C, Pace NR. 2000. Phylogenetic-comparative analysis of the eukaryal ribonuclease P RNA. *RNA* **6**: 1895-1904.

Gallardo F, Laterreur N, Cusanelli E, Ouenzar F, Querido E, Wellinger RJ, Chartrand P. 2011. Live cell imaging of telomerase RNA dynamics reveals cell cycle-dependent clustering of telomerase at elongating telomeres. *Molecular Cell* **44**: 819-827.

Gallardo F, Olivier C, Dandjinou AT, Wellinger RJ, Chartrand P. 2008. TLC1 RNA nucleo-cytoplasmic trafficking links telomerase biogenesis to its recruitment to telomeres. *EMBO J* **27**: 748-757.

Gibson DG, Young L, Chuang RY, Venter JC, Hutchison CA, 3rd, Smith HO. 2009. Enzymatic assembly of DNA molecules up to several hundred kilobases. *Nat Methods* **6**: 343-345.

Greider CW, Blackburn EH. 1987. The telomere terminal transferase of Tetrahymena is a ribonucleoprotein enzyme with two kinds of primer specificity. *Cell* **51**: 887-898.

-. 1989. A telomeric sequence in the RNA of Tetrahymena telomerase required for telomere repeat synthesis. *Nature* **337**: 331-337.

Gunisova S, Elboher E, Nosek J, Gorkovoy V, Brown Y, Lucier JF, Laterreur N, Wellinger RJ, Tzfati Y, Tomaska L. 2009. Identification and comparative analysis of telomerase RNAs from Candida species reveal conservation of functional elements. *RNA* **15**: 546-559.

Jiang J, Chan H, Cash DD, Miracco EJ, Ogorzalek Loo RR, Upton HE, Cascio D, O'Brien Johnson R, Collins K, Loo JA et al. 2015. Structure of Tetrahymena telomerase reveals previously unknown subunits, functions, and interactions. *Science* **350**: aab4070.

Jiang J, Miracco EJ, Hong K, Eckert B, Chan H, Cash DD, Min B, Zhou ZH, Collins K, Feigon J. 2013. The architecture of Tetrahymena telomerase holoenzyme. *Nature* **496**: 187-192.

Kuprys PV, Davis SM, Hauer TM, Meltser M, Tzfati Y, Kirk KE. 2013. Identification of telomerase RNAs from filamentous fungi reveals conservation with vertebrates and yeasts. *PLoS One* **8**: e58661.

Laterreur N, Eschbach SH, Lafontaine DA, Wellinger RJ. 2013. A new telomerase RNA element that is critical for telomere elongation. *Nucleic Acids Res* **41**: 7713-7724.

Lemieux B, Laterreur N, Perederina A, Noel JF, Dubois ML, Krasilnikov AS, Wellinger RJ. 2016. Active Yeast Telomerase Shares Subunits with Ribonucleoproteins RNase P and RNase MRP. *Cell* **165**: 1171-1181.

Lingner J, Hughes TR, Shevchenko A, Mann M, Lundblad V, Cech TR. 1997. Reverse transcriptase motifs in the catalytic subunit of telomerase. *Science* **276**: 561-567.

Lubin JW, Tucey TM, Lundblad V. 2012. The interaction between the yeast telomerase RNA and the Est1 protein requires three structural elements. *Rna* **18**: 1597-1604.

Maciejowski J, de Lange T. 2017. Telomeres in cancer: tumour suppression and genome instability. *Nat Rev Mol Cell Biol* **18**: 175-186.

Nakamura TM, Morin GB, Chapman KB, Weinrich SL, Andrews WH, Lingner J, Harley CB, Cech TR. 1997. Telomerase catalytic subunit homologs from fission yeast and human. *Science* **277**: 955-959.

Opresko PL, Shay JW. 2017. Telomere-associated aging disorders. *Ageing research reviews* **33**: 52-66.

Perederina A, Esakova O, Koc H, Schmitt ME, Krasilnikov AS. 2007. Specific binding of a Pop6/Pop7 heterodimer to the P3 stem of the yeast RNase MRP and RNase P RNAs. *RNA* **13**: 1648-1655.

Perederina A, Esakova O, Quan C, Khanova E, Krasilnikov AS. 2010. Eukaryotic ribonucleases P/MRP: the crystal structure of the P3 domain. *EMBO J* **29**: 761-769.

Peterson SE, Stellwagen AE, Diede SJ, Singer MS, Haimberger ZW, Johnson CO, Tzoneva M, Gottschling DE. 2001. The function of a stem-loop in telomerase RNA is linked to the DNA repair protein Ku. *Nat Genet* **27**: 64-67.

Podlevsky JD, Chen JJ. 2016. Evolutionary perspectives of telomerase RNA structure and function. *RNA Biol* **13**: 720-732.

Seto AG, Livengood AJ, Tzfati Y, Blackburn EH, Cech TR. 2002. A bulged stem tethers Est1p to telomerase RNA in budding yeast. *Genes & Development* **16**: 2800-2812.

Shippen-Lentz D, Blackburn EH. 1990. Functional evidence for an RNA template in telomerase. *Science* **247**: 546-552.

Singer MS, Gottschling DE. 1994. TLC1: template RNA component of Saccharomyces cerevisiae telomerase. *Science* **266**: 404-409.

Vasianovich Y, Wellinger RJ. 2017. Life and Death of Yeast Telomerase RNA. *J Mol Biol* **Online**: IN PRESS.

Watson JD. 1972. Origin of concatemeric T7 DNA. *Nat New Biol* **239**: 197-201.

Wellinger RJ, Wolf AJ, Zakian VA. 1993. Saccharomyces telomeres acquire single-strand TG1-3 tails late in S phase. *Cell* **72**: 51-60.

Wellinger RJ, Zakian VA. 2012. Everything you ever wanted to know about Saccharomyces cerevisiae telomeres: beginning to end. *Genetics* **191**: 1073-1105.

Wu RA, Upton HE, Vogan JM, Collins K. 2017. Telomerase Mechanism of Telomere Synthesis. *Annu Rev Biochem* **86**: 439-460.

Wu Y, Zakian VA. 2011. The telomeric Cdc13 protein interacts directly with the telomerase subunit Est1 to bring it to telomeric DNA ends in vitro. *Proceedings of the National Academy of Sciences of the United States of America* **108**: 20362-20369.

Zappulla DC, Cech TR. 2004. Yeast telomerase RNA: A flexible scaffold for protein subunits. *Proceedings of the National Academy of Sciences* **101**: 10024-10029.

Zappulla DC, Cech TR. 2006. RNA as a flexible scaffold for proteins: yeast telomerase and beyond. *Cold Spring Harbor Symposia on Quantitative Biology* **71**: 217-224.

Zappulla DC, Goodrich KJ, Arthur JR, Gurski LA, Denham EM, Stellwagen AE, Cech TR. 2011. Ku can contribute to telomere lengthening in yeast at multiple positions in the telomerase RNP. *Rna* **17**: 298-311.

**Table 1: Yeast strains for this study**

| **Name** | **Genotype** | **Reference** |
| --- | --- | --- |
| EBYH03 | *MATa* *can1-100 ura3-1 his3-11,15 leu2-3,112 trp1-1 bar1Δ::LEU2 tlc1Δ::NatMX* + pAZ1 | (Lemieux et al. 2016) |
| NLYH55 | *MATα ura3-52 lys2-801 ade2-101 trp1Δ63 his3Δ200 leu2Δ1 VR-ADE2-T tlc1Δ::NatMX cdc13Δ::KanMX* | (Laterreur et al. 2013) |
| NLYH59 | *MATα ura3-52 lys2-801 ade2-101 trp1Δ63 his3Δ200 leu2Δ1 VR-ADE2-T tlc1Δ::NatMX cdc13Δ::KanMX est1Δ::LoxP* | (Laterreur et al. 2013) |
| NLYH125 | *MATa leu2 trp1 ura3-52 prb- prc- pep4-3 EST1-Gly_6_-MYC_12_ FLAG_3_-MYC_12_- Gly_6_-EST2 tlc1Δ::NatMX HA_3_-POP1* | This study |
| NLYH128 | *MATa leu2 trp1 ura3-52 prb- prc- pep4-3 EST1-Gly_6_-MYC_12_ FLAG_3_-MYC_12_- Gly_6_-EST2 HA_3_-POP1 POP6-HA_3_:KMX* | This study |
| NLYH494 | *MATa leu2 trp1 ura3-52 prb- prc- pep4-3 EST1-Gly_6_-MYC_12_ FLAG_3_-MYC_12_- Gly_6_-EST2 tlc1Δ::NatMX* | (Lemieux et al. 2016) |
| YVL3493 | *MATa leu2 trp1 ura3-52 prb- prc- pep4-3 EST1-Gly_6_-MYC_12_ FLAG_3_-MYC_12_- Gly_6_-EST2* | (Lubin et al. 2012) |

**Table 2: Plasmids used in the study**

| **Name** | **Description** | **Reference** |
| --- | --- | --- |
| pAZ1 | *URA3, CEN, TLC1* | (Beeler et al. 1994) |
| pTLC1TRP | *TRP1, CEN, TLC1* | (Bah et al. 2004) |
| pTLC1-CS2Δ (pDel660) | *TRP1, CEN, tlc1Δ648-659* | (Seto et al. 2002) |
| pTLC1ΔP3 (pTLC1-ΔSL+IL) | *TRP1, CEN, tlc1 Δ602-642::GAATTC* | (Gunisova et al. 2009) |
| pTLC1-WT+1 | *TRP1, CEN, TLC1 600_601insC,643_644insG* | This study |
| pTLC1-WT+2 | *TRP1, CEN, TLC1 600_601insTC,643_644insGA* | This study |
| pTLC1-WT+3 | *TRP1, CEN, TLC1 600_601insATC,643_644insGAT* | This study |
| pTLC1-WT+6 | *TRP1, CEN, TLC1 600_601insATCTAG,643_644insCTAGAT* | This study |
| pTLC1-WT+9 | *TRP1, CEN, TLC1 600_601insATCTAGAAC  643_644insGTTCTAGAT* | This study |
| pTLC1-WT+12 | *TRP1, CEN, TLC1 600_601insATCTAGAACGTG 643_644insCACGTTCTAGAT* | This study |
| pTLC1-stemIVc+3 | *TRP1, CEN, TLC1589-590insCAC,660_661insGTG* | This study |
| pTLC1-P3NME1 | *TRP1, CEN, tlc1Δ601-643::NME1(31-76)* | (Lemieux et al. 2016) |
| pTLC1-P3NME1+3 | *TRP1, CEN, tlc1Δ601-643::ATC-NME1(31-76)-GAT* | This study |
| pTLC1-P3NME1+6 | *TRP1, CEN, tlc1Δ601-643::ATCTAG-NME1(31-76)-CTAGAT* | This study |
| pTLC1-P3NME1+9 | *TRP1, CEN, tlc1Δ601-643::ATCTAGAAC-NME1(31-76)-GTTCTAGAT* | This study |
| pTLC1-P3RPR1 | *TRP1, CEN, tlc1Δ601-643::RPR1(32-84)* | (Lemieux et al. 2016) |
| pVL1107 | *LEU2, CEN, CDC13-EST2* | (Evans and Lundblad 1999) |
| pNL09 | *URA3, HA_3_-POP1trunc [-715+2625] ins3xHA* | This study |
| pTLC1-10xMS2 | *TRP1, CEN, TLC1-10xMS2* | (Gallardo et al. 2011) |
| pTLC1-10xMS2-[WT+3] | *TRP1, CEN, TLC1-10xMS2 600*_*601insATC,643_644insGAT* | This study |
| pTLC1-10xMS2-CS2Δ | *TRP1, CEN, TLC1-10xMS2Δ648-659* | This study |
| pTLC1-10xMS2-ΔP3 (ΔL) | *TRP1, CEN, TLC1-10xMS2Δ602-642::GAATTC* | (Lemieux et al. 2016) |

**Figure Legends:**

**FIGURE 1.** Both the P3-like domain and the CS2 bulge sites on TLC1 are required for association of Est1 with the telomerase RNP. (*A*) Model showing the telomerase RNP and the MS2 binding stems-loops (dark red) at the 3’ end of the RNA. These will be bound by the MS2 core protein (MS2CP) fused with Protein A (ProA). A detailed view of Tlc1’s Stem IVc is shown on the left. The green nucleotides (nt 601-611) represent the CS2a element and the area within the green square is the P3-like domain. The blue nucleotides (nt 646-659) represent the CS2 bulge and the blue square represents the previously determined Est1 binding site. (*B*) Western blot after IP of telomerase components via the MS2 RNA stems and Protein A tagged MS2CP. Extracts from NLYH128 containing both a WT *TLC1* genomic copy and the indicated plasmid variants of the MS2-tagged *TLC1* were used. Lanes 1-5: 4% of the inputs. Lanes 6-10: 20% of the IP fractions. The corresponding tagged proteins were revealed using Myc- (Est1 and Est2) and HA-(Pop1, Pop6) specific antibodies. Strains of lanes 2 and 7 express a WT copy of *TLC1* on the plasmid. (*C*) Northern blot analysis of co-immunoprecipitated RNAs using IgG beads and extracts of NLYH128 (TAP-tagged MS2CP) transformed with the various *TLC1*-tagged plasmids. Lanes 1, 4, 7, 10, 13: IN, inputs (2.5%). Lanes 2, 5, 8, 11, 14: IP, immunoprecipitates (20%). Lanes 3, 6, 9, 12, 15: FT, flowthroughs (2.5%). The strain used for lanes 4-6 expresses an untagged version of *TLC1* on the plasmid; this RNA runs ahead of the tagged versions and is not visible on this part of the Northern. The top part of the blot was hybridized with a *TLC1-*specific probe and the bottom part was hybridized with a *NME1*-specific oligo.

**FIGURE 2.** The P3-like domain and the CS2 bulge form a tightly organized binding module. (*A*) Telomere length analyses in EBYH03 harboring the indicated *TLC1* alleles. Lanes 1-3: WT; lanes 4-6: tlc1Δ; lanes 7-9: tlc1-ΔP3; lanes 10-12: tlc1-[WT+1]; lanes 13-15: tlc1-[WT+2]; lanes 16-18: tlc1-[WT+3]; lanes 19-21: tlc1-*P3NME1*; lanes 22-24: tlc1-*P3NME1+3*. Schematics of stem IVc structures are represented on top. Red lines indicate the site of nucleotide insertions. Green lines represent the *NME1* stem-loop replacement within *TLC1*. +: strains carried a WT *TLC1* gene on a URA3 plasmid. Black triangles: growth of cells after loss of the WT *TLC1* gene for 30 and 110 generations. The blot was hybridized with telomere- and *CEN4-*specific probes. (*B*) Same telomere length analyses as in (*A*), except that longer insertions as indicated were analyzed (lanes 7-9: tlc1-[WT+6]; lanes 10-12: tlc1-[WT+9]; and lanes 13-15: tlc1-[WT+12]). Lanes with a ” + “, as well as the WT (lanes 1-3) and tlc1Δ (lanes 4-6) are as described above for (A).

**FIGURE 3.** SHAPE-reactivity results corroborate the predicted secondary structure model of the stem IVc arm of the different Tlc1 RNAs. SHAPE reactivity profiles of the stem IVc arm in the context of the WT, [WT+3] and ΔCS2 constructs. The regions in which the nucleotides are located in the predicted structures are indicated at the top. The relative reactivities higher than 0.4, between 0.4-0.2, and lower than 0.2 are shown in red, orange and black, respectively.

**FIGURE 4.** Est2 binding is stabilized upon Pop1 binding to the RNP via the P3-like domain. (A) Western blot (top) and Northern-IP (bottom) of the corresponding immunoprecipitation samples after pulldown of HA-tagged Pop1. Lane 1: NLYH494 strain expressing an untagged version of Pop1. Lanes 2-6: NLYH125 strain expressing HA_3_-Pop1 and the indicated *TLC1* alleles (except for lane 2 that has no *TLC1*). The indicated tagged proteins were revealed using Myc (Est2) or HA (Pop1) antibodies. Immunoprecipitated RNAs were visualized by hybridizing the northern membrane with *TLC1-* and *NME1-*specific probes. (*B*) Telomerase activity assay performed on same immunoprecipitated extracts as in (*A*). Indicated samples were treated with RNase to verify RNA-dependent activity (lanes marked with +). 0+^32^P: ^32^P-end-labeled substrate oligo. Loading ctrl: 12-nt labeled oligo. Relative activity/RNA ratios for the WT, WT+3 and CS2Δ samples were of 1.0, 1.1 and 1.0 respectively (n=2). The relative band intensities of the extension products were quantified and values were divided by the total RNA content verified by northern blots (data not shown). These were then corrected by HA_3_-Pop1 protein signal shown in (*A*), middle panel. The obtained values were adjusted relative to the WT Tlc1, which was set as 1.

**FIGURE 5.** The P3 domain is necessary for Est1-independent telomerase activity *in vivo*. (*A*) Telomere length analyses on DNA from *est1Δ-*cells expressing the *CDC13-EST2* fusion protein. Cells from the NLYH59 strain containing the indicated *TLC1* alleles (lanes 4-9 and 16-33) or in the absence of *TLC1* (lanes 10-15) were grown either with a WT copy of *TLC1* (pAZ1, + lanes) or for 110, 210, 310, 410 and 510 generations (black triangles) without *TLC1* (no pAZ1 plasmid). Lane 1 contains radiolabeled 1kb+ DNA ladder (MW in kb). Lanes 2-3: control strains bearing a WT copy of *TLC1* (pAZ1) and the *CDC13-EST2*-expressing plasmid. The blot was hybridized to a telomere-specific probe (pCT300) and a *CEN4*-specific probe as internal control.

(*B*) Telomere length analyses on DNA from *EST1wt-*cells also expressing the *CDC13-EST2* fusion protein. Cells from the NLYH55 strain containing the indicated *TLC1* alleles (lanes 4-8, 14-28) or in absence of *TLC1* (lanes 9-13) were grown either with a WT copy of *TLC1* (pAZ1, + lanes) or for 50, 110, 150 and 210 generations (black triangles). Lanes 2-3: control strains bearing a WT copy of *TLC1* (pAZ1) and the *CDC13-EST2*-expressing plasmid. Lane 1: 1kb+ DNA ladder (MW in kb). Blot was hybridized as in (A).

**Figure Legends for Spplementary Figures:**

**FIGURE S1.** MFold predicted secondary structures of the stem IVc arm of the Tlc1 WT, ΔP3, ΔCS2 and [WT+3] RNA variants. Blue: CS2 bulge (Est1 binding site). Green: CS2a element (Pop6/Pop7 binding). Red: mutated nucleotides leading to the indicated variant. Important sub-elements are indicated (Apical loop, 5’ and 3’ bulges, CS2 bulge, CS2 and CS2a elements).

**FIGURE S2.** TLC1 requires a very tight organization along Stem IVc to allow for telomere maintenance. Strain EBYH03 expressing the indicated *TLC1* alleles (lanes 2-4 and lanes 8-25) or bearing a *TLC1-*deletion (lanes 5-7) were grown in presence of a WT *TLC1* copy (+) or for 30 and 110 generations without WT *TLC1* (black triangles). The alleles of lanes 11-25 correspond to a Tlc1 RNA in which the P3-like domain was replaced with that of Nme1 (lanes 11-22) or Rpr1 (lanes 23-25) RNAs. Lanes 11-13: no insertion. Lanes 14-16: 3 base pairs insertion. Lanes 17-19: 6 base pairs insertion. Lanes 20-22: 9 base pairs insertion. Note that gDNA was lost for sample in lane 20. Lane 1: 1kb+ DNA ladder (MW in kb). Blot was hybridized as in Figure 1.

**FIGURE S3.** Telomere length of EBYH03 harboring a WT (lanes 1-3), empty (lanes 4-6) and a [stemIVc+3] (lanes 7-9) *TLC1-*containing plasmid. + and black triangles represent the same growth conditions as in Fig. 2. Schematic representations of the different variants are shown on top of the blot and red lines correspond to the site of the 3-bps insertion. Hybridization was carried as in Fig. 2.

**FIGURE S4.** Short term telomere length analyses in *est1Δ-*cells expressing the *CDC13-EST2* fusion protein. Cells from the NLYH59 strain containing the indicated *TLC1* alleles (lanes 2-4, 8-13) or in absence of *TLC1* (lanes 5-7) were grown either with a WT copy of *TLC1* (pAZ1, + lanes) or for 30 and 110 generations (black triangles). Lane 1 contain radiolabeled 1kb+ DNA ladder (MW in kb). Blot was hybridized to a telomere-specific probe (pCT300) and a *CEN4*-specific probe as internal control.

**FIGURE S5.** Telomere length analyses in the presence of a *CDC13-EST2* fusion and *EST1*. Cells from the NLYH55 strain containing the indicated *TLC1* alleles (lanes 4-9 and 16-33) or in absence of *TLC1* (lanes 10-15) were grown either with a WT copy of *TLC1* (pAZ1, + lanes) or for 110, 210, 310, 410 and 510 generations (black triangles) without pAZ1. Lane 1 contain radiolabeled 1kb+ DNA ladder (MW in kb). Lanes 2-3: control strains bearing a WT copy of *TLC1* (pAZ1) and the *CDC13-EST2*-expressing plasmid. The blot was hybridized as in Figure S3.
